# Position of the Voluntary Hospitals

**Published:** 1914-08-22

**Authors:** 


### Position of the Voluntary Hospitals.

### THE NECESSITY FOR CAUTION AND FORESIGHT.

The *Birmingham Daily Post* of the 15th inst. sounds a note of warning in regard to the depletion of the surgical and nursing staffs of voluntary hospitals, due to the war, based upon the effects it has had upon the General and Queen's Hospitals, Birmingham, and other voluntary hospitals in the district. It appears that at the General Hospital four nurses have gone to the Army, others to the Naval Nursing Service, and still more have left for the base hospital at the Birmingham University. The Territorial Hospital has absorbed a number of the surgical officers for duty, and although both nurses and doctors from the Territorial Hospital may be available for some duty at the General Hospital, the drain has taxed this institution sufficiently to make it necessary to issue a statement that only immediately urgent cases will be accepted for treatment and that no waiting list will in future be kept. At the Queen's Hospital many members of the staff who hold appointments in the Army Medical Service are now absent on military duty, and the committee find it impossible to continue the full work of the hospital, and are compelled to restrict the daily out-patient service to those cases requiring immediate attention. The hospital will be kept open for the relief of all accidents and cases of urgent illness, but the in-patient work must necessarily be diminished, though arrangements have been made to cause as little hardship as possible to the ordinary patients. The *Post* states that a private meeting has been summoned to consider the situation, and that it is probable that a central committee will be formed to consider if other than the present hospitals will be needed, and to examine the temporary hospitals which are being organised.

The *Post* quotes the Worcester Infirmary as "a typical example of the voluntary hospital position," so it is evidently unaware that the action its committee has taken in this connection to reduce the accommodation by sixty beds is only another example of the persistent policy of closing beds which many of its managers have advocated for a long time, and which we have consistently condemned.

This note of warning seems to us called for, having regard to the large number of hospitals in London and throughout the country which have offered to place many hundreds of beds at the disposal of the naval and military authorities for the accommodation of sick and wounded sailors and soldiers. If the war is to be prolonged, as it may be, or even if it extends through the winter into the spring, there can be no doubt that one of its effects will be to press hardly on the poorer residents, especially in towns, throughout the country; so that we may expect that, in addition to the average pressure of medical cases upon voluntary hospital beds, there must be great additional pressure. It is true, of course, that the modern tendency is to diminish medical beds and largely to increase those contained in voluntary hospitals for the accommodation of surgical cases. It may be argued that as a great number of the chief surgeons of the principal hospitals will be engaged on service in connection with the military, Territorial, and auxiliary home hospitals, the number of ordinary surgical cases may be considerably reduced so far as the voluntary hospitals are concerned, and that thus room may be found for more medical cases. Be this as it may, it is essential to provide that the ordinary and proper work and first duty of the voluntary hospitals shall be adequately fulfilled by supplying efficiently the claims of the civil population to in-patient treatment. We have dealt with this point at length in a leading article this week, and need not labour it here.

The point has not been overlooked by some, at any rate, of the metropolitan hospitals. Mr. G. Q. Roberts, the energetic secretary of St. Thomas's Hospital, has made a suggestion on behalf of the governors of that institution which is of practical value. It meets a point we have already urged—that it is important that an adequate number of beds should be made available in our chief hospitals, which are best equipped for the treatment of urgent surgical cases, enabling a great voluntary hospital of this type to place hundreds of beds at the disposal of the military and naval authorities for the reception of such cases arising in the time of war, whilst guaranteeing the civil population all the medical in-patient treatment which they may need for the reasons already explained. This suggestion is that each general hospital should have placed at its disposal accommodation in the emergency hospitals which certain wealthy people are fitting up at their own expense in many parts of the country, into which they shall admit cases from the general hospital who have passed the acute stage after a week or ten days in hospital, and are in a condition to be transferred to an emergency hospital. In this way the people who are organising private hospital accommodation should realise the truth, which is that they can best help soldiers and sailors by taking semi-convalescent patients, and so providing empty beds in the voluntary hospitals for the accommodation of the wounded needing active surgical care. We commend this suggestion to those whom it may specially concern, and hope that it will meet with the favourable consideration that it undoubtedly deserves.

### The Need for Open-air Hospitals.

Under this heading Dr. Robert Saundby, of Birmingham, consulting physician to the General Hospital, Professor of Medicine in the University, and Lieutenant-Colonel, R.A.M.C.T., writes an important letter, which appeared in the *Times* of the 15th inst., and is referred to in our Notes this week.

Dr. Robert Saundby makes three good points. First, that it may prove a very mistaken policy to attempt to convert buildings not designed for the accommodation of the sick into temporary hospitals at considerable expense—a plan which may cause a great deal of damage to the buildings themselves, and so entail much avoidable expense. Secondly, that experience in former wars, testified to by the Crown Prince of Prussia in a letter to Florence Nightingale, and, he might have added, much historical evidence, demonstrate that wounded men whose wounds may do badly in a hospital improve rapidly, and recover, on removal to an open-air building where they are exposed to abundance of fresh air and sunlight. Thirdly, in the Crimea, in the Civil War in America, and elsewhere, sick and wounded men did much better in open tents than in the hospitals. In Birmingham the Queen's Hospital has opened a roof ward, which has been working for some years, in which medical cases have been treated, summer and winter; and the same course has been followed at the General Hospital, the balconies of which have been enlarged so as to enable similar treatment to be extended to medical and surgical patients, with the best results. Dr. Saundby's just contention is that it would not only be more efficient, but cheaper, to put up suitable sheds in the parks and pleasure-grounds surrounding the buildings it is proposed to appropriate, and to use the mansions or public buildings as administration blocks. This plan would enable the patients to be kept on one floor, more or less in the open air, and it should not be difficult to arrange that the majority of such improvised buildings should be so constructed as to provide that all the wards should be open to the south.

Dr. Saundby's suggestion, it will be seen, has a very practical bearing upon some of our previous remarks; for if the suggestion is entertained it will simplify the establishment of a standard of requirement in the case of all mansions and public buildings offered for the reception of the sick and wounded throughout the country, and secure that they shall, when accepted, be converted, without any interference with the structure of existing buildings, into temporary hospitals—or, better still, into temporary semi-convalescent hospitals for the reception of sick and wounded men who have overcome the first shock of their disablement, and whose cases are such as to be likely to recover with increasing rapidity and success by being removed to one of these establishments where the open-air treatment can be carried on to the greatest advantage.

Of all the suggestions made, probably that of Dr. Robert Saundby is the most practical and valuable from every point of view. We commend its adoption to all who have it in their power to influence a decision in regard to it.

